# Recognition and Processing of Visual Information after Neuronavigated Transcranial Magnetic Stimulation Session

**DOI:** 10.3390/brainsci12091241

**Published:** 2022-09-14

**Authors:** Wiktoria Kasprzycka, Magdalena Ligia Naurecka, Bartosz Michał Sierakowski, Paulina Putko, Zygmunt Mierczyk, Grzegorz Chabik, Stanisław Dec, Stefan Gaździński, Rafał Rola

**Affiliations:** 1 Biomedical Engineering Centre, Institute of Optoelectronics, Military University of Technology, 00-908 Warsaw, Poland; 2 Department of Neurology, Military Institute of Aviation Medicine, 01-755 Warsaw, Poland

**Keywords:** TMS, fMRI, brain stimulation

## Abstract

Background: Transcranial magnetic stimulation (TMS) is a method of noninvasive and painless stimulation of the nervous system, which is based on Faraday’s law of electromagnetic induction. Over the past twenty years, the TMS technique has been deployed as a tool for the diagnosis and therapy of neurodegenerative diseases, as well as in the treatment of mental disorders (e.g., depression). Methods: We tested the inhibitory effects of repetitive TMS (rTMS) on reaction times to militarily relevant visual stimuli amidst distractors and on accompanying blood oxygenation level dependent (BOLD) signal functional magnetic resonance imaging (fMRI) in 20 healthy people. rTMS was applied over the visual cortices, V1, on both hemispheres with the inhibitory theta burst paradigm with the intensity of 70% of the active motor threshold fMRI in 20 healthy people. Results: Analysis of the reaction time to visual stimuli after using TMS to the V1 visual cortex revealed an increase in the number of incorrect recognitions, and the reaction time was from 843 to 910 ms. In the subgroup of participants (*n* = 15), after the stimulation, there were significant reductions of BOLD signal in blood flow within V1 cortices. Conclusions: The studies of reaction times after the rTMS revealed the inhibitory effect of rTMS on the reaction times and recognition performance of significant (military) objects in the visual field.

## 1. Introduction

Transcranial magnetic stimulation (TMS) is a relatively new technique that uses the short-duration pulses of a magnetic field to stimulate brain structures. TMS stimulates cortical tissues by using electromagnetic induction by discharging a strong, but short (up to 200 μs), electric current generated by the induction coil, which is placed over the examined cortical area [1,2]. A single-phase or two-phase current pulse generates a rapidly changing and short-lived magnetic field punctually perpendicular to the coil plane. The magnetic field induces an electric field in brain tissue and depolarizes neurons, which eventually modifies their activity directly at the site of stimulation and in connected areas of the brain [3,4,5]. The coil’s magnetic field does not induce current flow in skin and bone due to their relatively high electrical resistance. This makes TMS a painless and well-tolerated method [6]. The only inconvenience for the patient may be the sound of the discharge or contraction of the directly stimulated facial muscles.

Since the approval of TMS in the treatment of depression, there has been considerable interest in using it in other neuropsychiatric diseases such as post-traumatic stress, obsessive compulsive disorder, and schizophrenia [7,8,9,10,11]. Previous research indicates that TMS is also a useful tool for restoring motor functions after a stroke, and it can also be used in neurorehabilitation [11,12]. Even though TMS has been used for therapeutic purposes since the second half of the 1990s, the biophysical basis underlying the stimulation of neurons in the central nervous system is still under investigation. Research has shown that different neurological effects can be observed, including excitatory or inhibitory effects at targeted brain structures, depending on the frequency of magnetic pulse generation used. Hence, the elicited effect is largely dependent on the frequency of stimulation and the site of the brain application. rTMS is considered to produce an excitatory effect when using high-frequency protocols (≥1 Hz) or intermittent theta-burst stimulation, whereas an inhibitory effect is achieved by using low-frequency TMS protocols (≤1 Hz) or continuous theta-burst stimulation [4,13,14]. A single TMS session changes the excitability of the cortex for between 10 and 60 min, using most protocols [15]. The short-term inhibition effect of the individual regions of the visual cortex may play a significant role in the alteration of visual perception [16]. Stimulation of the primary visual cortex can result in visual impressions, such as sparkling or flashing lights, called phosphenes. Repeated rTMS sessions are used for long-term changes in synaptic plasticity. This type of stimulation is used to achieve a lasting therapeutic effect. Zhang et al. after stimulation of primary visual cortex in patients with major depressive disorder, he achieved a reduction in unnatural activity from the visual to the pre/subgenual anterior cingulate cortex, and subjects reported fewer symptoms [17]. The intensity of the TMS pulse must be at a level that allows effective current induction in the nervous tissue. For reasons of patient safety and comfort, this should be the minimum level at which response from the nervous system is received.

Most often, the stimulation of the primary visual cortex is related to its retinoscopic structure and the induction of phosphenes in various areas of the visual field [18,19]. According to the best of our knowledge, only a few groups of researchers have used transcranial magnetic stimulation directly in the area of the primary visual cortex for the study of visual information perception and processing, among others, for research on blindsight [20,21] or word recognition [22]. However, these studies were limited to a single magnetic pulse (or just a few pulses) in different locations, and the TMS effect was investigated in a very short period of time after stimulation, in the order of milliseconds.

The study aimed to assess the effect of TMS on the recognition of significant visual stimuli from several minutes to an hour after a session of 400 pulses delivered to the primary visual cortex. The study was designed to evaluate the functional effects of TMS on visual perception. We hypothesized increases of reaction times after application of inhibitory TMS bursts, as well as lower changes in blood oxygentation level dependent (BOLD) signal after the inhibitory TMS pulses.

## 2. Materials and Methods

### 2.1. Participants and Safety of Research

We tested 20 healthy people (11 women and 9 men aged 21 to 39 years). All of the participants were informed about the nature of the study, completed a safety questionnaire based on the one recommended by The Safety of TMS Consensus Group [23], and expressed their written consent to participate in the study. The survey received a positive opinion from the MIAM Bioethics Committee on 29.11.2016 (decision No. 16/2016). All of the participants were neurologically healthy and did not have any history of epileptic seizures. They were also asked to eat a light breakfast and refrain from consuming caffeinated drinks on the day of the study. The research took place in the morning hours.

**Figure 1:**
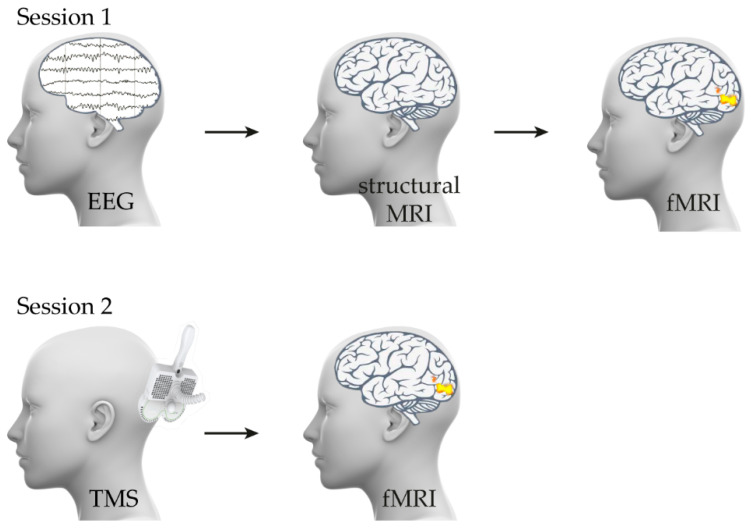
Scheme of experiment.

The study protocol assumed two Independent sessions with an interval of about a month. This was to avoid memorizing the sequences and objects presented during the study (Figure 1). During the first session, participants initially had routine electroencephalography (EEG) to exclude persons with epileptiform activity in the brain and T1 structural magnetic resonance imaging (MRI) scan to find regions of interest (ROI) for stimulation, taking into account the anatomical variability of the cortex. The participants underwent imaging in a 3T GE Discovery 750 W magnetic resonance tomograph, with a 70-cm-wide bore, using a body transmit coil for excitation and an eight-channel receive coil. Anatomical scans were obtained by using high-resolution FSPGR-BRAVO (TR/TE/TI = 8.484/3.268/450 ms), at a resolution of 1 × 1 × 1.2 mm^3^, covering the entire brain. In all of the participants, the routine EEG was normal and structural MRI did not show any changes that would be a contraindication to further participation in the study.

### 2.2. fMRI: Task and Stimuli

The same study of the reaction time to the visual stimulus (attributable to military objects) was executed without TMS and immediately after TMS in two independent sessions (Figure 1). The visual stimuli were presented on an LCD screen at the resolution of 1920 × 1080 pixels (InroomViewingDevice, NordicNeuroLab, Bergen, Norway). The stimuli were back-projected on the screen and viewed through a system of mirrors mounted inside the head coil. The reaction time was assessed by using protocols about perceiving and recognizing significant visual (military) stimuli in the visual field.

**Figure 2:**
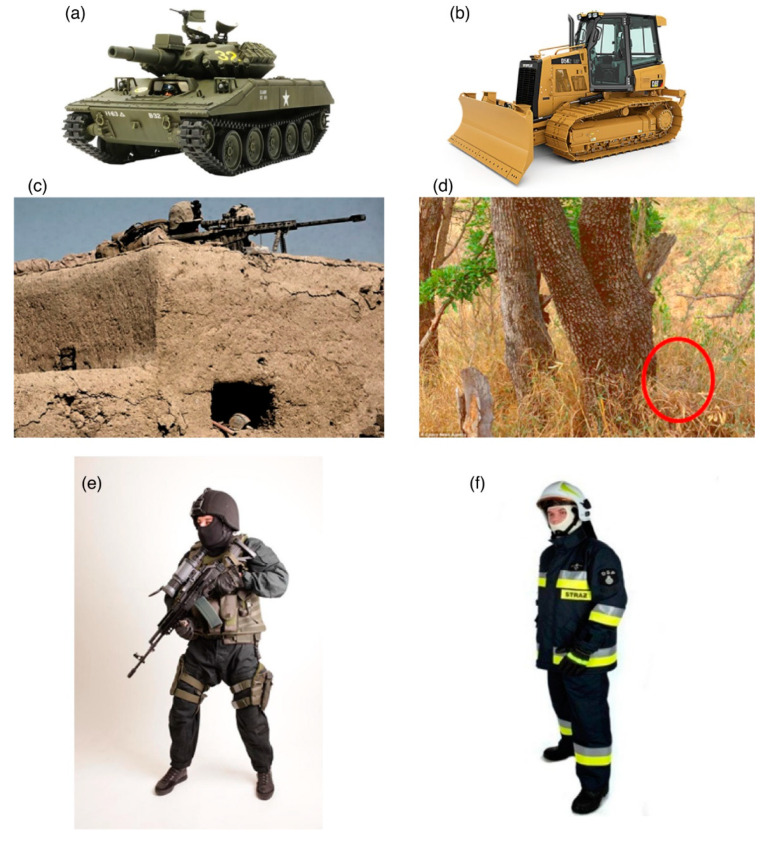
Examples of stimuli associated with the military (**a**,**c**,**e**) and distractors (**b**,**d**,**f**).

The subjects underwent a visual stimulation procedure with visual stimuli by using an event-related paradigm: 32 of the stimuli presented potentially dangerous objects and 48 served as distractors (Figure 2). Each image was presented for 100 ms among irrelevant distractors that were, however, related semantically with the important stimulus against the black field of view. The participants had to react to the stimuli with the right hand for military-relevant stimuli and with the left hand for the other stimuli by using NNL ResponseGrip (NordicNeuroLab, Bergen, Norway). A series of figures were presented in a pseudo-random sequence in each of the quarters of the field of view in the randomly selected time intervals of 2 to 5 s (see Figure 2). Significant stimuli were presented in the entire field of view. The essence of stimulating the right and left hemispheres was the inhibition of visual perception in the full field of view. The optimization of time intervals between presented images was done by using the free OptSeq program (http://surfer.nmr.mgh.harvard.edu/optseq, accessed on 2 December 2020). After the picture was presented, the subject had to press the right button for pictures associated with the military, and the left button in other cases. The responses were recorded and were later used to assess the level of performance. The time between figure presentation and correct answer (by pressing the relevant button) was recorded as the reaction time. Two or more errors were exclusionary, and one participant was excluded. The entire study was performed with the recording of BOLD signal changes by using fMRI (3T GE Discovery 750 W). Functional images were acquired with echo planar images with the following parameters: TE = 30 ms, TR = 2000 ms, 35 slices acquired in an ascending interleaved order, 3.125 × 3.125 × 3.5 mm resolution, 0.5 mm gap between slices, and covering the entire head. Each run consisted of 305 volumes and was preceded by seven dummy scans. The same set of stimuli (pictures) was used at both fMRI sessions, but they appeared in random sequence and in randomly selected time intervals. In the numerical analysis of fMRI, we used standard parameters.

The latency between the presentation of the relevant stimulus and the time of pressing the appropriate key was measured. This latency was a measure of the speed of recognition and psychomotor reactions to an important stimulus. The latencies of the recognition of important stimuli without and after the TMS stimulation session were compared.

### 2.3. TMS Stimulation

Transcranial magnetic stimulation was performed with the DuoMAG XT-100 stimulator with DuoMAG 70BF figure of eight butterfly coil with 2 × 70 mm windings and the neuronavigation BrainSight TMS system (Brainbox Ltd, Cardiff, UK). Initially, the T1 scans of each subject were uploaded to the BrainSight TMS system, adjusted, and remodeled into a three-dimensional model. Then, the neuronavigation system was calibrated and validated on the subject. The M1 cortex representing the cortical area for the dominant hand was then localized. The EMG Ag, pre-gelled electrodes were placed over the first dorsal interosseus muscle of the dominant hand. In order to attenuate interference in the analyzed signal, a 1 Hz high-pass filter and a 5 kHz low-pass filter were used.

**Figure 3:**
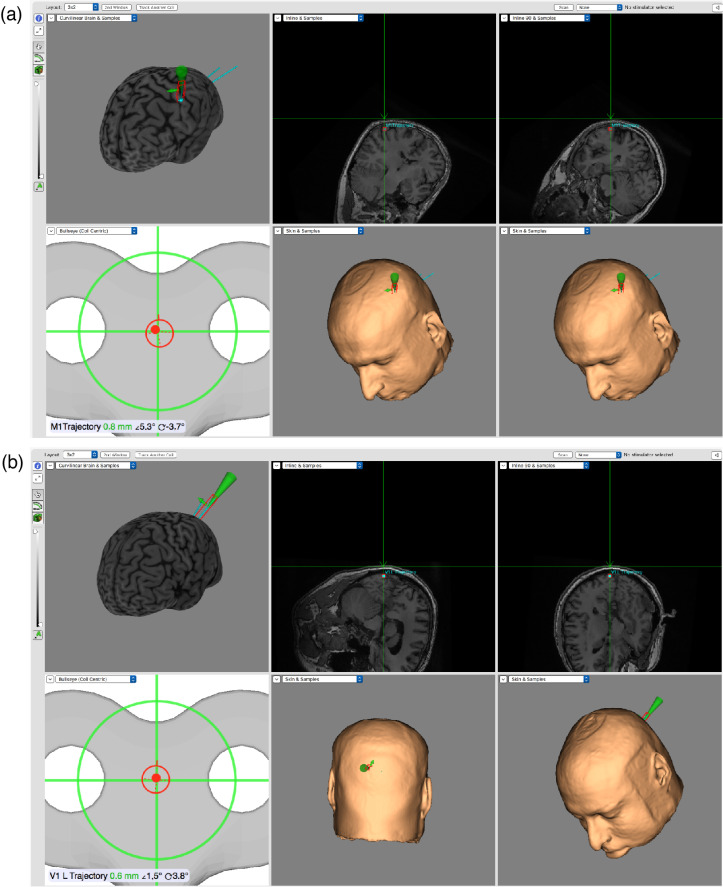
Localization of the hotspot for the motor threshold (**a**) and for the V1 cortex (**b**) with neuronavigation.

After the localization of the hotspot (Figure 3a), the resting motor threshold (RMT) was assessed with the adaptive PEST algorithm by using software from the Medical University of South Carolina [24]. The method relies on adaptive parameter estimation by sequential testing (PEST) to determine RMT based on previously selected intensities and their success or failure. RMT is defined as the minimum stimulation intensity that motor-evoked potential (MEP) is significant in at least half of the trials. A trial is considered a success when MEP has minimal value of 50 μV (usually 50–100 μV) [25,26]. Thanks to neuronavigation, it was easy to find a hotspot and achieve a high MEP for all participants in almost all trials. The authors decided to take a slightly higher threshold as a successful trial and set it at 120 μV. Peak-to-peak MEP amplitudes were determined relative to the base signal measured for 20 ms prior to the TMS pulse. The method assumes that the distribution of parameters takes the form of a cumulative Gaussian curve. On this basis, the midpoint of the curve is estimated, which is taken as the true threshold value (RMT). The PEST method significantly reduces the number of attempts made to effectively determine RMT, increasing the safety of the procedure [27].

The orientation of the coil over the scalp was chosen according to the principle that the cortex responds most strongly to fields oriented in a manner perpendicular to the cortical layers. Coil orientation was posterior to anterior, 45 degrees relative to the sagittal plane, with the handle pointing backward. Due to the greater stability of the movement threshold than the threshold of the appearance of phosphenes, which are more variable and dependent on the menstrual cycle phase, among others, and different in conditions of closed eyes and conditions of the presentation of visual stimuli. In this study, stimuli significant in meaning for the participants were previously unknown and presented quickly. The visual excitability threshold in such a paradigm would be complicated to quantify.

Afterward, the stimulating coil was targeted toward the V1 region (Figure 3b) of each hemisphere. After finding the right spot, the stimulating coil and the subject’s head were fixed, and the procedure of rTMS was initiated. The theta burst suppression (TBS) protocol was a series of 3 pulses at 50 Hz and power of 70% of resting motor threshold, repeated 200 times with a frequency of 5 Hz for each subject. Left and right V1 cortices were stimulated. Coil orientation was anterior to posterior, 45 degrees relative to the sagittal plane, with the handle pointing up. Immediately after the stimulation, the subject was transferred to the fMRI scanner.

### 2.4. Data Analysis

Data were processed and analyzed by using Statistical Parametric Mapping (SPM12, Welcome Department of Cognitive Neurology, London, UK, http://www.fil.ion.ucl.ac.uk/spm, accessed on 15 December 2020) implemented in a Matlab 2017a environment. For the same patient, the same structural study was used for two studies to normalize to the MNI atlas space, thus minimizing inaccuracies in the analysis. The contrasts of interest were first computed at the individual level to identify the cerebral regions significantly activated by the stimuli (pictures). Significant cerebral activations for the critical contrasts were then examined at the group level in random-effect analyses by using paired t-tests with the statistical threshold set at *p* < 0.05 (FWE corrected at the cluster level), similar to Fierro et al. [28].

## 3. Results

Analysis of the accuracy of recognition of significant visual stimuli in terms of meaning showed that, after the transcranial magnetic stimulation on the visual cortex V1, there was a significantly higher number of no recognition of significant images (military objects) in comparison to the test before the application of TMS (χ^2^ test *p* < 0,05). The analysis of reaction times for the correct recognition of significant visual stimulus (military objects) indicated an increase in the recognition time in 16 participants, on average from 843 ± 98 ms to 910 ± 133 ms (Student’s *t*-test *p* < 0,05).

**Figure 4:**
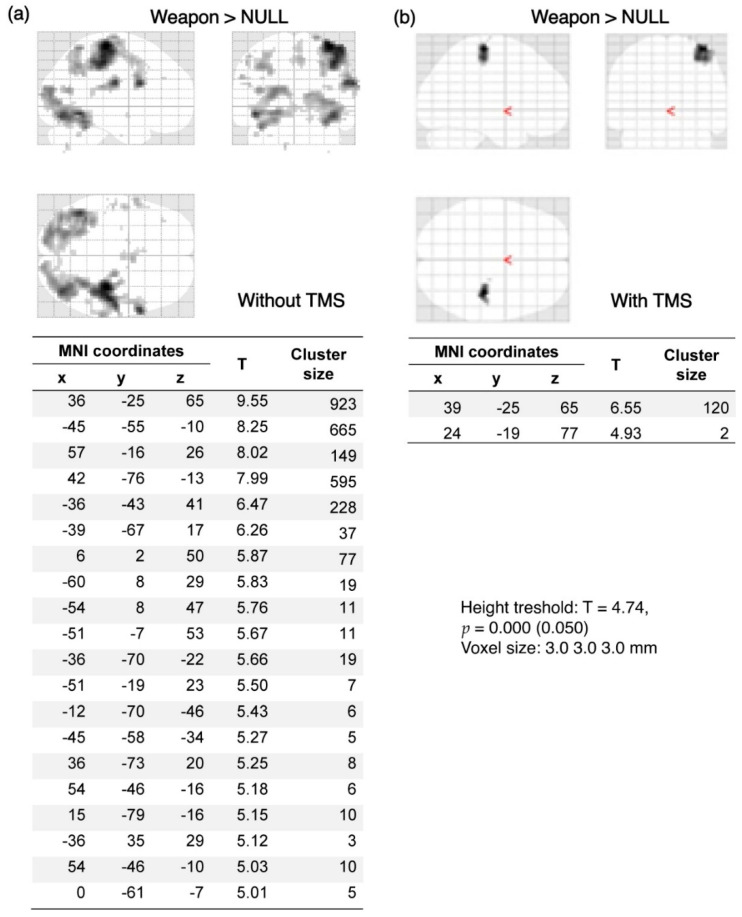
Decrease of BOLD signal activation in response to the visual stimulus of significant significance without (**a**) and after (**b**) using TMS. Tables show 3 local maxima more than 8.0 mm apart. Visibly clear decrease of the BOLD signal in the occipital region after applying TMS. “Weapon” denotes activations evoked by images related to the military compared to brain activation when no stimuli were presented (NULL).

**Figure 5:**
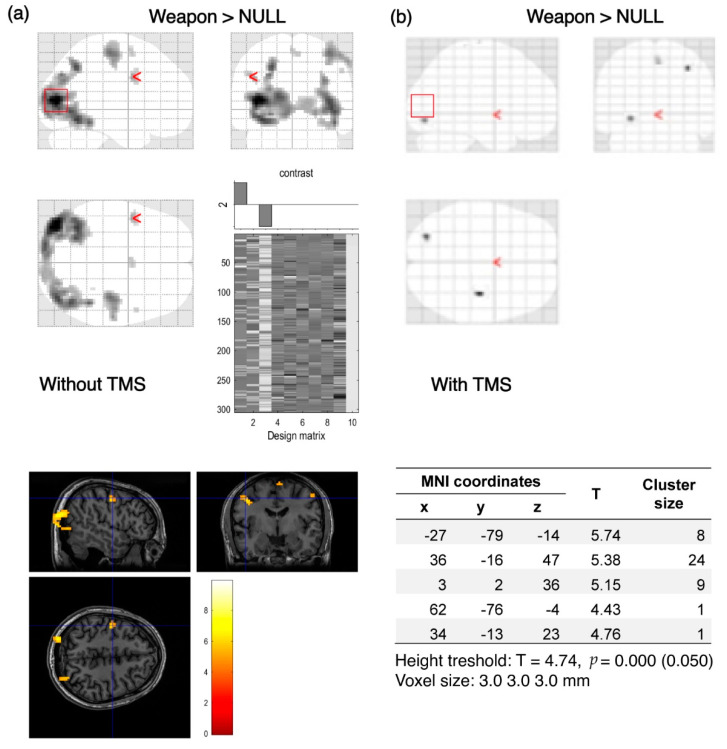
Decrease of BOLD signal activation in response to the visual stimulus of significant significance before (**a**) and after (**b**) using TMS. Visible clear decrease of BOLD signal in the occipital region (red squares) after applying TMS. Table shows 3 local maxima more than 8.0 mm apart. Visibly clear decrease of BOLD signal in the occipital region after applying TMS. “Weapon” denotes activations evoked by images related to the military compared to brain activation when no stimuli were presented (NULL).

**Figure 6:**
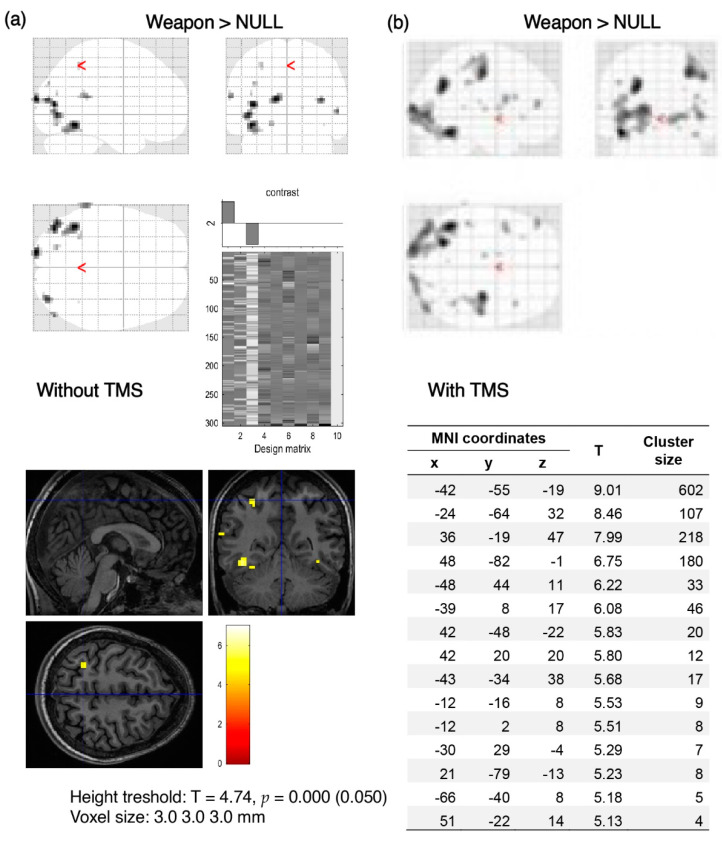
One of two cases of the subjects that shows a decrease in activation of the BOLD signal (*n* = 2) in response to the visual stimulus of significant significance without TMS (**a**) compared to visual stimulus after transcranial magnetic stimulation (**b**). The visibly clear increase in the BOLD signal in the occipital region after applying TMS. Table shows 3 local maxima more than 8.0 mm apart. “Weapon” denotes activations evoked by images related to the military compared to brain activation when no stimuli were presented (NULL).

The results of the analyses illustrate the comparison of activation before and after the application of TMS. In the majority of participants (*n* = 15), after using TMS there was a visible reduction in activation, mostly in the visual cortex (Figure 4 and Figure 5). These figures show data from individual volunteers. Two participants showed increased activation in the visual cortex after using TMS, as shown in Figure 6, which was probably related to the change in basal blood flow or some compensating brain mechanisms to maintain the level of performance (see [29]). Changes in BOLD signal in the remaining three participants were not statistically significant.

Group analyses involving all participants (*n* = 20) and only those participants who demonstrated decreases in the BOLD signal (*n* = 15) did not show statistically significant results (*p* < 0.05, FWE). The latter findings likely result from spatial differences in changes of the BOLD signal among participants. Similarly, the BOLD responses to military-related and irrelevant stimuli were not different, probably due to differences in strategies used by different participants to distinguish military-related from irrelevant stimuli (Figure 2).

## 4. Discussion

It is assumed that the effect of a single pulse of TMS is the interruption of the current activity of neurons. Several authors suggest adding random noise to the signal, which can alter the image processing stream by reducing objective performance or consistency between the accuracy and visibility ratings [20,30], or even signal fading (reduced visibility).

Single-pulse TMS used to the occipital cortex (the lower-left visual field) during visual working memory (VWM) consolidation affects the number of items that are stored, or their precision [31]. The results of this study suggest that the activity of the visual cortex directly stimulated by TMS is disturbed mainly by the number of elements effectively stored, but also, although to a lesser extent, affects the quality of VWM localization. Observed disruptive effects were largest when pulses of TMS were applied very early during the retention interval (up to 100 ms), growing smaller or disappearing completely at longer intervals. TMS also reduced swap errors for objectives contralateral to the stimulated hemisphere and improved retinotopically specific color–location. However, studies [32] on the application of 10 Hz TMS to the lateral occipital cortex and the marginal gyrus show an improvement in cognitive abilities, whereas other scientists using similar protocols indicated their deterioration [33,34].

High-frequency rTMS stimulation in the dorsolateral prefrontal cortex in young men improved their attention control [35], but in this study the TBS protocol successfully produced the opposite effect, confirming the possibility of influencing the visual cortex and the processing of visual information by altering the frequency of the TMS pulses.

The aim of this research was to assess the negative impact of TMS on the reaction time and recognition of important visual stimuli (related to military objects). We used two techniques: measurements of recognition times, and activation of the regional blood flow in the visual cortex after the inhibitory (theta burst suppression) rTMS on V1 cortices. Analysis of reaction time to visual stimuli after stimulation with TBS indicates an increase in the amount of wrong reconnoitering, and a decrease in regional BOLD signal flow activation (also called brain activity) after significant visual stimulus for at least 1 h after the stimulation. The reaction times of the correct reconnoitering of the visual stimulus associated with military objects indicated that the TMS prolonged the reaction time by an average of 70 ms. These studies confirm that noninvasive transcranial magnetic stimulation can decrease the brain activity of a healthy person. However, our results point to presence of some compensating brain mechanisms to maintain cognitive performance (reaction time) that can be observed as increases in the activated areas (areas of increased BOLD signal). Such compensatory mechanisms were described in the literature, e.g., [29].

These increases in reaction times and changes in accompanying brain activation effects may be caused by an interruption of visual information processing. Regardless of their physiological mechanisms (interruption of neuronal activity within the V1 cortex, or interruption of information flow to the V2 and V3 cortices), such effects can have significant negative consequences for people who work in a visual context and have to recognize and quickly react to significant stimuli.

Moreover, recent studies show that patients with major depressive disorder who underwent visual stimulation and rTMS in the V1 region reported fewer symptoms. This coincided with a decrease in unnatural activity from the visual to the pre/subgenual anterior cingulate cortex seen on fMRI [17].

The main limitation of this study was the small number of young volunteers. Research on a larger and more diverse group in terms of age would allow us to better understand the impact of rTMS on the correct identification of military and civilian objects and reaction times. The parameters used for stimulation have a huge impact on the observed effects because increasing the number of impulses in the motor cortex may enhance the rTMS effect [36] or, on the contrary, weaken it [37]. The major limitation of using functional MRI is that the method assumes the use of simple stimuli (pictures) that activates the same, well-defined brain regions with high probability. To maintain military relevance of the stimuli, they needed to be complex; therefore, their analysis likely involved various (not always the same) cognitive processes that activated different brain regions. Therefore, it seems that the use of fMRI to assess changes in brain activation to real-life military equipment is limited with the current methodology. Furthermore, we cannot exclude that the participants employed strategies in identifying the presented objects. Also, the BOLD signal correlation with EEG amplitudes in alpha and beta bands was not performed, which would allow for a more detailed analysis of the data. Finally, we did not use sham TMS excitations, so some contribution of the sound effects accompanying TMS on reaction times and BOLD signal cannot be completely excluded.

## 5. Conclusions

TMS enables the stimulation of neurological reactions, e.g., processes of stimulation or a weakening of neurological activity. It can be concluded from the conducted research that TBS protocol is an inhibitory stimulus that, in most cases, inhibits/reduces the activation of the visual cortex and reaction time by about 8% (from 843 ± 98 to 910 ± 133 ms), under the influence of a visual stimulus connected with the reconnoitering of military objects. Consequently, there is an increase in incorrect recognitions and an overlong reaction time. Analysis of reaction time to visual stimuli after applying TMS to the V1 visual cortex showed an increase in the number of wrong recognitions and the reaction time, by about 70 ms. It also showed that the presence of a significant visual stimulus increased the activation of the primary V1 and secondary regions of the visual and parietal cortex.

## Data Availability

Not applicable.
